# Recent progress in multidisciplinary treatment for patients with esophageal cancer

**DOI:** 10.1007/s00595-019-01878-7

**Published:** 2019-09-18

**Authors:** Masayuki Watanabe, Reiko Otake, Ryotaro Kozuki, Tasuku Toihata, Keita Takahashi, Akihiko Okamura, Yu Imamura

**Affiliations:** Department of Gastroenterological Surgery, The Cancer Institute Hospital of Japanese Foundation for Cancer Research, 3-8-31 Ariake, Koto-ku, Tokyo, 135-8550 Japan

**Keywords:** Esophageal cancer, Multidisciplinary treatment, Esophagectomy, Chemoradiotherapy

## Abstract

Esophageal cancer is one of the most aggressive gastrointestinal cancers. This review focuses on eight topics within the multidisciplinary approach for esophageal cancer. As esophagectomy is highly invasive and likely to impair quality of life, the development of less invasive strategies is expected. Endoscopic resection (ER) of early esophageal cancer is a less invasive treatment for early esophageal cancer. A recent phase II trial revealed that combined ER and chemoradiotherapy (CRT) is efficacious as an esophagus-preserving treatment for cT1bN0 squamous cell carcinoma (SCC). Esophagectomy and definitive CRT are equally effective for patients with clinical stage I SCC in terms of long-term outcome. For locally advanced resectable cancers, multidisciplinary treatment strategies have been established through several clinical trials of neoadjuvant or perioperative treatment. Minimally invasive esophagectomy may improve the outcomes of patients and CRT is a curative-intent alternative to esophagectomy. CRT with 50.4 Gy radiotherapy combined with salvage surgery is a promising option to preserve the esophagus. Induction chemotherapy followed by esophagectomy may improve the outcomes of patients with locally advanced unresectable tumors. Immune checkpoint inhibitors are effective for esophageal cancer, and their introduction to clinical practice is awaited.

## Introduction

Esophageal cancer is one of the most aggressive of all gastrointestinal malignancies. The overall 5-year survival rate ranges from 15 to 25% worldwide and it is the sixth leading cause of cancer-related deaths of men [1]. In Japan, the 5-year survival rate of patients with esophageal cancer diagnosed between 2006 and 2008 was 37.2% (36.0% of men and 43.9% of women) [2]. The two major histologic subtypes of esophageal cancer are squamous cell carcinoma (SCC) and adenocarcinoma (AC). It is well-known that the incidence of both subtypes varies among geographic areas: SCC has a higher prevalence in East Asia, Eastern and Southern Africa, and Southern Europe, whereas AC is much more common in North America and other parts of Europe [3]. In Japan, SCC is the main histologic subtype, accounting for approximately 90% of cases, but the incidence of AC is increasing [4].

Although esophagectomy remains the mainstay of treatment for esophageal cancer, it is very invasive and associated with a high incidence of morbidity and mortality [5]. Moreover, postoperative symptoms such as appetite loss, early satiety, dysphagia, aspiration, and reflux can impair the patients’ quality of life [6]. Therefore, a less-invasive alternative to esophagectomy is being sought, especially for patients with early-stage disease. Unfortunately, esophageal cancer is frequently found at an advanced stage when surgery alone cannot achieve cure. For such cases, a multidisciplinary treatment strategy is required to achieve cure. This review summarizes recent progress in the multidisciplinary treatment of esophageal cancer.

## Methods

In this narrative review, we focus on eight topics: endoscopic resection for early esophageal cancer, treatment selection for cStage I (T1bN0) SCC, neoadjuvant treatment for locally advanced resectable SCC, multidisciplinary treatment strategy for locally advanced resectable AC, recent progress in surgical technique, definitive chemoradiotherapy (CRT) and salvage esophagectomy, conversion surgery for initially unresectable SCC, and immune checkpoint blockade for esophageal cancer.

### Endoscopic resection for early esophageal cancer

Endoscopic resection (ER) is a less invasive treatment for early esophageal cancer than other treatment modalities such as esophagectomy and definitive CRT. The indication for ER is decided based on the risk of lymph node metastasis. Lymph node metastasis seldom arises from carcinoma in situ or tumors confined within the lamina propria mucosae; therefore, ER can be curative for these tumors. The reported incidence of lymph node metastasis in patients with SCCs invading the muscularis mucosa and those with minimal invasion to the submucosal layer (< 200 μm below the muscularis mucosa) was 9.3% and 19.6%, respectively [7]. These lesions are considered to be a relative indication for ER.

The indication for ER in AC patients is still based on the same criteria as used for SCC, although there is little information on the risk of lymph node metastasis from early-stage AC. A recent Japanese multicenter retrospective study found that lymphatic invasion, the existence of a poorly differentiated component, and a lesion size > 30 mm, were the factors associated with lymph node metastasis in patients with mucosal or submucosal ACs [8]. In that study, tumors limited to within 500 μM of submucosal invasion had a low incidence of lymph node metastasis unless they had the risk factors mentioned above. Based on these results, the clinical features of early-stage esophageal AC seem to be more similar to those of early gastric cancer [9] than to those of esophageal SCC.

Recently, Muto et al. reported the results of the Japan Clinical Oncology Group (JCOG) 0508 trial, which was a multi-institutional, single-arm phase II study to evaluate the efficacy and safety of combined ER and CRT in patients with cT1bN0M0 SCC [10]. The study enrolled patients with cT1bN0M0 SCCs with tumor size ≤ 5 cm and ≤ 3/4 circumference. They divided the patients into three groups based on the pathologic results: Group A included 74 patients with pT1a with negative resection margin and no vascular invasion; Group B included 87 patients with pT1b with negative resection margin or pT1a with vascular invasion; and Group C included 15 patients with pT1b with positive resection margin. The Group A patients were followed up without any additional treatment; the Group B patients underwent prophylactic CRT consisting of 41.4 Gy/23 fractions (fr) irradiation to the field, including locoregional lymph nodes, combined with 5-fluorouracil + cisplatin (CF); and the Group C patients underwent definitive CRT consisting of 50.4 Gy/28 fr with a 9-Gy boost to the primary site. The 3-year overall survival rate of the Group B patients, the primary endpoint, was 90.7% [90% confidence interval (CI) 84.0–94.7], suggesting that the combined ER and CRT strategy is efficacious as esophagus-preserving treatment for cT1bN0M0 SCC.

### Treatment selection for cStage I (cT1bN0) SCC

Esophagectomy is the standard treatment for cStage I SCC, worldwide. The current National Comprehensive Cancer Network Guideline (Version 2.2019) in the USA states that esophagectomy is the standard treatment for cT1bN0 SCC and that definitive CRT may be an appropriate option for patients who decline surgery [11]. According to the European Society for Medical Oncology Clinical Practice Guidelines, surgery is the treatment of choice for limited disease (cT1–T2N0M0), irrespective of the histologic subtype, although definitive CRT is recommended for patients not willing to undergo esophageal surgery or who are medically unfit for major surgery [12]. The current Japanese guideline states that the selection between surgery and CRT should be made after assessing the patient’s surgical tolerability [13].

In Japan, efforts are being made to establish an esophagus-sparing treatment for T1bN0 SCC using definitive CRT, even for patients who are fit for surgery. The JCOG9708 trial, a phase II study of definitive CRT for clinical stage I SCC, revealed 2- and 5-year overall survival rates of 93% and 76%, respectively, which are comparable to those of esophagectomy [14]. The results of the JCOG0502 trial, which is a parallel-group trial of esophagectomy versus CRT for patients with clinical Stage I esophageal carcinoma, have been reported recently [15]. Although this study had a randomized part, it was terminated because of poor patient accrual. In the comparison of the non-randomized part, the 3- and 5-year overall survival rates of CRT group were 93.1% (95% CI 87.9–96.1) and 85.5% (95% CI 78.9–90.1), respectively, while those of the surgery group were 94.7% (95% CI 90.8–97.0) and 86.5% (95% CI 81.0–90.5), respectively. The adjusted hazard ratio (HR) was 1.082 (95% CI 0.674–1.640), suggesting the non-inferiority of CRT against esophagectomy.

### Neoadjuvant treatment for locally advanced resectable SCC

The long-term outcomes of surgery alone for patients with locally advanced SCC are unsatisfactory. Many trials on adjuvant or neoadjuvant therapy have been conducted to investigate efficacy. Table 1 shows the clinical trials that are the basis of current standard treatment for locally advanced, resectable esophageal SCC. The JCOG9204 trial, which compared surgery alone versus surgery followed by postoperative chemotherapy using the CF regimen, revealed that postoperative chemotherapy extended the disease-free survival of patients with node-positive clinical stage II/III SCC [16]. The JCOG9907 trial compared preoperative CF followed by esophagectomy versus esophagectomy followed by postoperative CF for patients with clinical stage II/III SCC [17]. The trial clarified that the overall survival of the preoperative chemotherapy group was significantly better than that of the postoperative chemotherapy group. Based on these results, neoadjuvant chemotherapy with CF is the current standard treatment for cStage II/III SCC in Japan. For patients who undergo upfront surgery, postoperative CF is recommended if the pathologic examination detects lymph node metastasis [13]. Currently, a three-arm phase III trial comparing CF versus docetaxel versus cisplatin plus 5-fluorouracil (DCF) versus CRT using CF as preoperative therapy for locally advanced esophageal cancer (JCOG1109) is ongoing [18].

**Table 1:** Clinical trials that are the basis of current standard treatment for locally advanced resectable esophageal squamous cell carcinoma

Study name (year)	Histologic subtype^a^	Treatment arms^b^	Main results^c^	*P* value
JCOG9204 (2003)	SCC	Surgery alone Surgery + CF	5-year DFS 45% 5-year DFS 55%	0.037
JCOG9907 (2012)	SCC	Surgery + CF CF + surgery	5-year OS 43% 5-year OS 55%	0.04
CROSS (2012)	SCC/AC	Surgery alone CRT + surgery	Median OS 24.0 M Median OS 49.4 M	0.003
JCOG1109 (ongoing)	SCC	CF + surgery DCF + surgery CF-RT + surgery	–	–

^a^*SCC* squamous cell carcinoma, *AC* adenocarcinoma

^b^*CF* cisplatin + 5-fluorouracil, *CRT* chemoradiotherapy, *DCF* docetaxel + cisplatin + 5-fluorouracil, *RT* radiotherapy

^c^*DFS* disease-free survival, *OS* overall survival

In Western countries, preoperative CRT is the gold standard for patients with locally advanced resectable SCC, based on the results of the CROSS trial, which compared surgery alone with neoadjuvant CRT consisting of 40 Gy irradiation combined with carboplatin plus paclitaxel followed by surgery [19, 20]. Although the CROSS regimen provided survival benefits to patients with both histologic subtypes, the survival benefit was more evident in those with SCC than in those with AC.

In the neoadjuvant CRT arm of the CROSS trial, a complete response was seen pathologically in the resected specimen from 49% of patients with SCC and 23% of patients with AC [19]. Based on these high complete response rates, the necessity of standard esophagectomy after neoadjuvant CRT has been questioned, especially for patients who responded sufficiently to neoadjuvant CRT. The preSANO trial was a prospective, multicenter, diagnostic cohort study to establish the accuracy of detection of residual disease after neoadjuvant CRT with different diagnostic approaches [21]. This study revealed that clinical evaluation with endoscopic ultrasonography, bite-on-bite biopsies, and fine-needle aspiration of suspicious lymph nodes was adequate for the detection of locoregional residual disease, with PET–CT for the detection of interval metastases. Based on the result, surveillance using this combination is being assessed in a phase III trial (SANO trial) [22].

### Multidisciplinary treatment strategy for locally advanced resectable AC

The standard treatment for locally advanced AC in Japan has not yet been established because of the limited number of cases of AC. In Western countries, several important clinical trials have demonstrated the efficacy of neoadjuvant or perioperative therapy versus surgery alone (Table 2). The OEO2 trial, in which 66% of the patients had AC, revealed that neoadjuvant CF extended overall survival more effectively than surgery alone [23]. The Medical Research Council Adjuvant Gastric Infusional Chemotherapy (MAGIC) trial, in which 11% of patients had junctional esophageal cancer and 14% had lower esophageal AC, compared three cycles of epirubicin, cisplatin, and 5-fluorouracil (ECF) before and after surgery with surgery alone [24]. The 5-year overall survival rates of the perioperative chemotherapy group versus the surgery alone group were 36% versus 23%, respectively (*P* = 0.009). Notably, the efficacy of perioperative chemotherapy was independent of the tumor location. The FNCLCC-FFCD trial compared perioperative CF with surgery alone and found that the overall survival of the perioperative chemotherapy group was significantly better than that of the surgery alone group [25]. The CROSS trial also demonstrated the efficacy of neoadjuvant CRT in patients with AC [19, 20]. The FLOT4 trial was a randomized, phase II/III trial that evaluated the safety and efficacy of the docetaxel-based triplet FLOT (fluorouracil plus leucovorin, oxaliplatin and docetaxel) as perioperative chemotherapy for locally advanced, resectable AC [26]. Phase III of this study revealed that overall survival was significantly better in the FLOT group than in the ECF/ECX group (HR, 0.77; 95% CI 0.63–0.94).

**Table 2:** Clinical trials of multidisciplinary treatment strategy for locally advanced resectable esophageal adenocarcinoma

Study name (year)	Histologic subtype^a^	Treatment arms^b^	Main results^c^	*P* value
OEO2 (2002)	SCC, AC	Surgery alone CF + surgery	5-year OS 17% 5-year OS 23%	0.03
MAGIC (2006)	Esophagogastric AC	Surgery alone ECF + surgery + ECF	5-year OS 23% 5-year OS 36%	0.009
FNCLCC-FFCD 9703 (2011)	Esophagogastric AC	Surgery alone CF + surgery (+CF)	5-year OS 24% 5-year OS 38%	0.02
CROSS (2012)	SCC, AC	Surgery alone CRT + surgery	Median OS 24.0 M Median OS 49.4 M	0.003
FLOT4 (2017)	Esophagogastric AC	ECF or ECX + surgery + ECF or ECX FLOT + surgery + FLOT	Median OS 35 M Median OS 50 M	0.012
Neo-AEGIS (ongoing)	Esophagogastric AC	CRT + surgery (CROSS) Peri CT (ECF, ECX, EOF, EOX)	–	–
ESOPEC (ongoing)	Esophagogastric AC	CRT + surgery (CROSS) FLOT + surgery + FLOT	–	–
TOPGEAR (ongoing)	Esophagogastric AC	Peri CT (ECF/ECX) Peri CRT (ECF + CRT + surgery + ECF/ECX)	–	–

^a^*SCC* squamous cell carcinoma, *AC* adenocarcinoma

^b^*CF* cisplatin + 5-fluorouracil, *ECF* epirubicin + cisplatin + 5-fluorouracil, *CRT* chemoradiotherapy, *ECX* epirubicin + cisplatin + xeloda, *FLOT* 5-fluorouracil + leucovorin + oxaliplatin + taxotere, *DCF* docetaxel + cisplatin + 5-fluorouracil, *Peri CT* perioperative chemotherapy

^c^*DFS* disease-free survival, *OS* overall survival

Several phase III trials comparing perioperative chemotherapy versus neoadjuvant chemoradiotherapy for AC are ongoing. The Neo-AEGIS trial is a phase III trial comparing the modified MAGIC regimen (ECF/ECX or EOF/EOX) versus neoadjuvant CRT with the CROSS regimen [27]. The ESOPEC trial is a randomized controlled trial comparing perioperative chemotherapy with the FLOT regimen versus neoadjuvant CRT with the CROSS regimen [28]. The TOPGEAR trial is an international phase III trial comparing perioperative ECF alone versus ECF with CRT. These trials may change the standard treatment for locally advanced resectable esophageal AC in the near future [29].

### Recent progress in surgical techniques

Minimally invasive esophagectomy (MIE) using thoracoscopy and/or laparoscopy is being performed increasingly, worldwide. According to the Annual Report from The Japanese Association for Thoracic Surgery, MIE was adopted for 1036 patients (51.3%) with superficial cancer and 1734 patients (42.0%) with advanced cancer in Japan during 2015 [30]. Two randomized controlled trials have been conducted to evaluate the efficacy of MIE compared with traditional open esophagectomy (OE). The TIME trial compared MIE versus OE for cT1–3, N0–1, M0 esophageal cancer [31]. The primary outcome was pulmonary infection within the first 2 weeks and during the whole stay in hospital. There were 56 patients assigned to the OE group and 59 patients assigned to the MIE group. Pulmonary infection developed in the first 2 weeks in 16 (29%) and 5 (9%) of the OE and MIE group patients, respectively [relative risk (RR), 0.30; 95% CI 0.12–0.76; *P* = 0.005], while 19 (34%) and 7 (12%), respectively, suffered pulmonary infection in hospital (RR, 0.35; 95% CI 0.16–0.78; *P* = 0.005). The hospital stay was significantly shorter for the MIE group than for the OE group. The short-term QOL was better in MIE group than in OE group. Three-year follow-up of this trial revealed no difference in disease-free or overall 3-year survival for OE and MIE [32]. The MIRO trial compared the transthoracic open procedure (open procedure) versus a hybrid minimally invasive procedure using a laparoscopic approach (hybrid procedure) in patients undergoing Ivor-Lewis esophagectomy [33]. The primary endpoint was intraoperative or postoperative complications of Clavien–Dindo Grade II or higher within 30 days. There were 105 patients assigned to undergo the hybrid procedure and 104 assigned to undergo the open procedure group. A total of 37 patients (36%) in the hybrid procedure group and 67 (64%) in the open procedure group suffered a major intraoperative or postoperative complication (odds ratio, 0.31; 95% CI 0.18–0.55; *P* < 0.001). The 3-year survival rate was 67% (95% CI 57–75) for the hybrid procedure group and 55% (95% CI 45–64) for the open procedure group. Although the difference was not significant, the 3-year overall survival tended to be better in the hybrid procedure group than in the open procedure group.

In Japan, patient registration for the National Clinical Database (NCD) commenced in January, 2011. The NCD project contains the records of ≥ 95% of the operations performed by general surgeons in Japan [34]. Several studies evaluated the short-term outcomes of MIE and OE, using the NCD data. The first study analyzed postoperative complication and mortality after OE and MIE for 5,354 patients who underwent esophagectomy in 2011 [5]. Overall morbidity was significantly higher in the MIE group than in the OE group (44.3% vs. 40.8%, *P* = 0.016). In particular, the incidences of anastomotic leakage and reoperation within 30 days were significantly higher in the MIE group than in the OE group (14.9% and 8.0%, *P* = 0.016; vs. 12.5% and 5.6%, *P* = 0.001). A subsequent study evaluated 9,584 patients with thoracic esophageal cancer who underwent esophagectomy in 2011–2012 [35]. In that study, one-by-one matching between the OE and MIE groups based on estimated propensity scores for each patient was carried out. After propensity score matching, the operative time was significantly longer in the MIE group than in the OE group (526 ± 149 vs. 461 ± 156 min, *P* < 0.001), while blood loss was remarkably less in the MIE group than in the OE group (442 ± 6121 vs. 608 ± 591 ml, *P* < 0.001). Both the incidence of atelectasis and the population of patients who required more than 48 h of postoperative mechanical ventilation were significantly lower in the MIE group than in the OE group, whereas the incidence of pneumonia was similar in the two groups. The reoperation rate within 30 days was significantly higher in the MIE group than in the OE group (7.0 vs. 5.3%, *P* = 0.004). The latest analysis included 24,233 esophagectomies for esophageal cancer performed between 2012 and 2016 [36]. That study analyzed the influence of preoperative treatment and surgical procedures on short-term outcomes. The total surgery-related mortality rate was significantly lower in the MIE group than in the OE group (1.7% vs. 2.4%, *P* < 0.001). MIE was superior or equivalent to OE in terms of the incidence of postoperative morbidities and surgery-related mortality, regardless of the type of preoperative treatment. MIE performed with no preoperative treatment was associated with a lower incidence of pulmonary morbidities, prolonged ventilation for more than 48 h, unplanned intubation, surgical site infection, and sepsis. However, reoperation within 30 days was more frequent in the MIE group than in the OE group among patients who underwent esophagectomy without preoperative treatment. Currently, a phase III trial comparing MIE versus OE is ongoing in Japan [37].

Mediastinoscopy-assisted transhiatal esophagectomy (MATHE) represents another MIE option, which has the potential benefit of decreasing pulmonary complications by avoiding one-lung ventilation [38]. MATHE was considered as a less invasive option and the insufficient mediastinal lymphadenectomy was overcome by inserting a mediastinoscope from the cervical wound using a single-incision laparoscopic surgery device to enable upper mediastinal lymph node dissection under a sufficient mediastinoscopic view with carbon dioxide insufflation [39]. Fujiwara et al. reported that the median number of mediastinal lymph nodes resected by the novel MATHE was 21, achieving an R0 resection rate of 95% [40]. Postoperative pneumonia developed in 4 of 60 patients (6.7%). Mori et al. reported a robotic-assisted technique of MATHE [41]. In their retrospective comparison, the number of harvested mediastinal lymph nodes was comparable in the robotic-assisted MATHE and conventional transthoracic esophagectomy [42].

Robotic surgical systems were developed to overcome the technical limitations of conventional minimally invasive surgery. Robotic-assisted MIE (RAMIE) was introduced in 2003 [43], since when several case series have reported its safety with good oncological outcomes [44, 45]. The ROBOT trial was a single-center randomized controlled trial comparing RAMIE with open thoracic esophagectomy (OTE) [46]. The primary endpoint was the percentage of overall surgery-related postoperative complications of Clavien–Dindo Grade II or higher. In that study, 112 patients were randomized to undergo either RAMIE or OTE. The overall complication rate was significantly lower in the RAMIE group than in the OTE group (59% vs. 80%, *P* = 0.02). Notably, both pulmonary complications (32% vs. 58%, *P* = 0.005) and cardiac complications (22% vs. 47%, *P* = 0.006) were significantly lower in the RAMIE group than in the OTE group. Although this study provided evidence for the use of RAMIE to improve the short-term outcomes of esophagectomy, the benefits of RAMIE over conventional MIE are not entirely clear and its cost-effectiveness is frequently challenged. Further research is required to clarify the advantages of RAMIE over MIE.

### Definitive chemoradiotherapy and salvage esophagectomy

Definitive CRT with curative intent is an alternative to esophagectomy for patients who decline or are unfit for surgery. For patients with clinical Stage I SCC, the JCOG0502 trial demonstrated the survival non-inferiority of definitive CRT against esophagectomy, already mentioned [15]. However, it should be noted that 21 of the 159 patients (13.2%) in the CRT group underwent salvage surgery. The JCOG9906 trial was a phase II trial that explored the efficacy and safety of CRT for patients with cStage II/III esophageal SCC [47], while the JCOG9907 compared preoperative chemotherapy followed by surgery versus surgery followed by postoperative chemotherapy for similar patients [17]. Both trials had almost identical eligibility criteria. The JCOG1406-A was an exploratory analysis using pooled data from two prospective trials: JCOG9906 and JCOG9907 [48]. Overall survival was significantly better in the preoperative chemotherapy group, followed by the esophagectomy group, than in the CRT group (adjusted HR 1.72; 95% CI 1.19–2.50). Unlike for patients with cStage I SCC, for patients with cStage II/III SCC, CRT is not standard treatment, but an alternative to multidisciplinary treatment with esophagectomy as the main modality.

Although salvage esophagectomy can provide a chance of cure when definitive CRT fails, it is associated with high morbidity and mortality rates. We reported previously that a high radiation dose ≥ 60 Gy was a significant predisposing factor to postoperative pulmonary complications [49]. The INT0123 RTOG 94-05 trial compared the locoregional control, survival, and toxicity associated with CRT using high-dose (64.8 Gy) versus standard-dose (50.4 Gy) radiation therapy for patients with esophageal cancer [50]. The higher radiation dose did not increase survival or locoregional control, and the standard radiation dose for definitive CRT is considered to be 50.4 Gy. Based on this result, a phase II study of concurrent CRT at the dose of 50.4 Gy with elective nodal irradiation (modified RTOG regimen) for stage II–III esophageal carcinoma was conducted [51]. A complete response was achieved in 36 of 51 patients (70.6%), and the 1- and 3-year overall survival rates were 88.2% and 63.8%, respectively. There was no mortality of the 8 patients who underwent salvage esophagectomy. The JCOG0909 trial was the single-arm phase II study to verify the efficacy and safety of CRT with the modified RTOG regimen followed by salvage treatment for locoregional failure in patients with cStage II/III esophageal SCC [52]. The complete response rate was 59%, the 3-year overall survival rate was 74.2%, and the 3-year esophagectomy-free survival was 63.6%. Major complications of Clavien–Dindo Grade III or more developed in 5 (20.2%) of 25 patients who underwent salvage esophagectomy and 1 (4.0%) died. These results show that CRT with the modified RTOG regimen is a promising option to preserve the esophagus.

Definitive CRT is the standard treatment for locally advanced, unresectable esophageal cancer. Ohtsu et al. reported the efficacy of definitive CRT consisting of concurrent 60 Gy radiotherapy with CF for patients with T4 and/or M1 lymph node (LYM) esophageal cancer [53]. Complete response was achieved in 18 (33%) of 54 patients, 9 of whom had T4 disease. The JCOG9516 trial was a phase II study of CRT for T4 and/or M1 LYM [54]. The objective response rate was 68.3%, the complete response rate was 15%, the median survival time was 10 months, and the 2-year survival rate was 31.5%. The JCOG0303 trial was a randomized phase II/III study comparing standard CF (arm A) versus daily low-dose CF (arm B) with concurrent 60 Gy radiotherapy for SCC patients with T4 and/or unresectable regional lymph node metastasis [55]. There was no difference in the toxicities between the groups. The median and the 3-year overall survivals were 13.1 months and 25.9%, respectively, for arm A, and 14.4 months and 25.7% for arm B. Although the low-dose CF regimen was expected to be more effective and less toxic than the standard CF, there was no obvious advantage of the low-dose CF regimen over the standard CF regimen. The KDOG0501 trial was a phase I/II trial to evaluate the safety and efficacy of definitive CRT with the DCF regimen (DCF-R) for locally advanced SCC [56, 57]. Phase I suggested that DCF-R was tolerable and active in patients with advanced esophageal cancer [56]. Phase II investigated the efficacy of RCF-R for patients with T4 and/or M1LYM. The total radiation dose was initially 61.2 Gy, but this was lowered to multiple-field irradiation with 50.4 Gy to decrease esophagitis and late toxicity [57]. The clinical complete response rate was 52.4% overall: 33.3% in the 61.2 Gy group and 60.0% in the 50.4 Gy group. The median overall survival was 29.0 months and the 3-year survival rate was 43.9%. Although major toxicity of Grade 3 or more was frequently observed, DCF-R is suggested to be a promising regimen for patients with locally advanced, unresectable SCC.

### Conversion surgery for initially unresectable SCC

The availability of effective chemotherapy regimens and the development of innovational surgical techniques have resulted in more cases that would once have been considered unresectable, now being resectable following treatment [58]. This strategy is known as conversion therapy and the surgery performed is called conversion surgery. Although conversion therapy is commonly performed for patients with colorectal cancer, the clinical significance of such a strategy for patients with esophageal cancer remains unclear. A multicenter phase II trial assessing the safety and efficacy of chemoselection with induction chemotherapy using DCF and subsequent conversion surgery for initially unresectable locally advanced esophageal SCC was conducted in Japan [59]. Treatment started with induction DCF, followed by conversion surgery if resectable, or by CRT if unresectable. Twenty of the 48 patients enrolled (41.7%) underwent conversion surgery and R0 resection was achieved in 19 (39.6%). In addition to the patients who underwent conversion surgery, clinical complete response after CRT was confirmed in 4 patients (8.3%) and the 1-year overall survival rate of the enrolled patients was 67.9%. These results suggest that chemoselection with DCF induction chemotherapy followed by conversion surgery is a promising strategy for patients with locally advanced, unresectable SCC. Currently, a phase III trial comparing this strategy versus definitive chemoradiotherapy plus salvage surgery (JCOG1510 trial) is ongoing [60].

### Immune checkpoint blockade for esophageal cancer

The development of the immune checkpoint blockade has launched a new era of immunotherapy. Programmed death-1 (PD-1), together with its ligand (PD-L1) inhibit the response of T cells to tumor cells [61]. An open-label, single-arm, multi-center phase II trial was conducted to assess the safety and activity of nivolumab, an anti-PD-1 antibody, monotherapy for metastatic esophageal SCC in patients refractory to standard chemotherapy [51]. Eleven of the 64 patients enrolled (17%) had an objective response (partial response rate of 15.6% and complete response rate of 1.6%) and the median overall survival was 10.8 months (95% CI 7.4–13.3). A phase III trial comparing nivolumab alone and docetaxel or paclitaxel (NCT02569242) is ongoing. The results from the esophageal cancer cohort of KEYNOTE-028, a multicohort phase IB trial of pembrolizumab, another anti-PD1 antibody, in patients with PD-L1 positive advanced solid tumors have also been reported [62]. The study included patients with either SCC or AC, for whom standard chemotherapy failed. Among the 23 patients enrolled, 18 (78%) had SCC. The objective response rate was 28% (5/18) for patients with SCC and 40% (2/5) for those with AC. The median duration of response was 15 months and the median overall survival was 7.0 months. The KEYNOTE-181 trial was a phase III study comparing pembrolizumab versus the investigator’s choice chemotherapy as second-line therapy for patients with advanced/metastatic SCC or AC of the esophagus or Siewert type I AC of the esophagogastric junction [63]. Among the 628 patients enrolled, 401 had SCC and 222 had a combined positive score (CPS) ≥ 10. Pembrolizumab was superior to chemotherapy for overall survival when the CPS ≥ 10 (median 9.3 vs. 6.9 months; HR 0.69; 95% CI 0.52–0.93; *P* = 0.0074). A phase III trial comparing chemotherapy plus pembrolizumab versus chemotherapy alone as first-line therapy for advanced esophageal cancer (NCT03189719) is ongoing [64].

## Conclusion

Esophageal cancer is challenging to treat and requires a multidisciplinary approach to improve the outcomes. The results of further and ongoing clinical trials will contribute to establishing the most appropriate interdisciplinary strategy for each stage of each histologic subtype.
