# Correction to: Recent progress in multidisciplinary treatment for patients with esophageal cancer

**DOI:** 10.1007/s00595-019-01952-0

**Published:** 2020-01-10

**Authors:** Masayuki Watanabe, Reiko Otake, Ryotaro Kozuki, Tasuku Toihata, Keita Takahashi, Akihiko Okamura, Yu Imamura

**Affiliations:** Department of Gastroenterological Surgery, The Cancer Institute Hospital of Japanese Foundation for Cancer Research, 3-8-31 Ariake, Koto-ku, Tokyo, 135-8550 Japan

## Correction to: Surgery Today (2020) 50:12–20 https://doi.org/10.1007/s00595-019-01878-7

The article Recent progress in multidisciplinary treatment for patients with esophageal cancer, written by Masayuki Watanabe, Reiko Otake, Ryotaro Kozuki, Tasuku Toihata, Keita Takahashi, Akihiko Okamura, Yu Imamura, was originally published Online First without Open Access. After publication in volume 50, issue 1, page 12–20 the author decided to opt for Open Choice and to make the article an Open Access publication. Therefore, the copyright of the article has been changed to © The Author(s) 2020 and the article is forthwith distributed under the terms of the Creative Commons Attribution 4.0 International License (https://creativecommons.org/licenses/by/4.0/), which permits use, duplication, adaptation, distribution and reproduction in any medium or format, as long as you give appropriate credit to the original author(s) and the source, provide a link to the Creative Commons license, and indicate if changes were made.

The original article has been corrected.

